# Brain asymmetry as minimization of free energy: a theoretical model

**DOI:** 10.1098/rsos.240465

**Published:** 2024-07-31

**Authors:** Giorgio Vallortigara, Giuseppe Vitiello

**Affiliations:** ^1^ Centre for Mind/Brain Sciences, University of Trento, Piazza della Manifattura 1, Rovereto, Trento I-38068, Italy; ^2^ Department of Physics 'E.R. Caianiello', University of Salerno, Via Giovanni Paolo II, 132, Fisciano (Salerno) I-84084, Italy

**Keywords:** asymmetry, lateralization, free energy

## Abstract

The asymmetry between the left and right sides seems to be a general principle of organization of the nervous systems in Bilateria, providing the foundations for a plethora of leftward and rightward biases in behaviour as documented in species ranging from *Caenorhabditis elegans* nematodes to humans. Several theories have been put forward to account for the existence and maintenance in the evolution of the asymmetric organization of the brain at both individual and population levels. However, what is missing in theorizing about the evolution of brain asymmetry is an overarching general hypothesis that may subsume all different aspects of current models. Here, we tried to provide an overarching general framework based on the energy and free-energy minimization principle, which proved so valuable in other areas of neuroscience. We found that at the individual level the antisymmetric singlet configuration realizes the lowest energy state of the system, whereas at the group level, the spontaneous emergence of directional asymmetry arises as a consequence of the minimization of the free energy of the system, which guarantees its stability and equilibrium. We thus argue that the various phenomenological aspects of brain asymmetry that have been captured in biology—e.g. sparing of neural tissue, control of unitary motor responses and, at the population level, evolutionarily stable strategies described by mathematical games theory—may be thought of as the manifestation of a more general principle of energy minimization generating, among others, asymmetry of the brains.

## 1. Introduction

Having a left or right bias may appear inconvenient, if not a nuisance, for biological organisms negotiating their environment. Molecules, of course, have chirality, but for the kind of large-size objects organisms are dealing with (including other organisms), we should expect the physical world to be indifferent to left and right: a predator (a prey, a companion) may appear in principle on either side, and, apart from accidents in developmental processes (so-called fluctuating asymmetries [1]) the morphology of Bilateria (animals with bilateral symmetry) should be expected to be symmetrical.

However, it is now firmly established that these biological organisms exhibit several forms of asymmetries, most notably in their nervous systems and in their behaviour (reviews in [2,3]).

Vertebrates have been extensively investigated in this regard. In general, it seems that the left side of the vertebrate brain is specialized for the control of well-established patterns of behaviour under ordinary and familiar circumstances, whereas the right hemisphere is the primary seat of emotional arousal, specialized for detecting and responding to unexpected stimuli in the environment [4].

Brain asymmetries probably date back to the Cambrian period [5]. Examples are apparent in various consistent biases favouring one or the other side for either motoric or sensory activities. For instance, animals with laterally placed eyes (and sometimes also those with large binocular overlaps) systematically use their left or right eye in selecting prey, emitting agonistic responses, responding to predators, sleeping, or responding to social signals (for general reviews including also different sensory modalities see [6–10] and for recent results involving causal mechanisms see [11–14]). Evidence for behavioural and brain asymmetries is widespread and not limited at all to only vertebrates, ranging from the nematodes *Caenorhabditis elegans* [15] to human beings [16]. Thus, we should expect that asymmetries provide some important fitness benefits, and different hypotheses have been put forward. To understand these hypotheses, however, it should be noted that asymmetries in the brain and behaviour may occur at the individual and population levels and that these two levels raise different questions as to their benefits and costs [17].

An individual animal may be asymmetric in a particular direction; say, the right side of the brain can be specialized for recognizing faces [18]. A fitness benefit of such an asymmetry at the individual level can be associated with an increase in neural capacity because specialization of one side of the brain for a cognitive function, such as recognizing faces, can avoid useless duplication of functions between the two hemispheres, leaving space for other functions in the homologous regions in the other hemisphere (a decrease in redundancy would have, of course, important costs, as in the case of a lesion or other pathology affecting only one hemisphere). Given that the brain represents only approximately 2% of the body weight of an adult human but accounts for approximately 20% of the calories consumed by the body, sparing of such a metabolic costly tissue would represent a benefit.

Sparing of neural tissue is certainly not the only possible advantage of asymmetry at the individual level [19]. Other advantages have been suggested, and there is empirical evidence suggesting that they all may have played some role in the evolution of asymmetry. For example, for animals with laterally placed sense organs, as most Bilaterians, that have quite independent representations of the left and right visual fields, ensuring a unique course of action towards stimuli perceived in each field can be accomplished only by making one side of the brain, depending on tasks and circumstances, fully in control of overt behaviour, maybe through inhibitory connections between the two sides of the brain [20]. Furthermore, lateralization would offer the advantage of allowing simultaneous and parallel processing of information on the two sides of the brain—in chicks and in other species, it has been proved that having an asymmetrical brain improves performance in dual tasks while leaving unaffected the ability to do each task separately [21].

Then comes the second issue, namely the advantage of population lateralization. Individuals could be lateralized without any need to show the same direction of lateralization as a group. Even though examples of individual-level lateralization have been observed in animal behaviour (with a 1 : 1 distribution of left and right phenotypes), what is striking is that in most vertebrates and also in invertebrates lateralization of brain and behaviours is observed at the level of group, with a majority (varying in magnitude but always showing a significant departure from 50%) of individuals favouring one side (e.g. right hemisphere for face processing, left hemisphere for language production, a right hemisphere for spatial functions, left hemisphere for control of the dominant hand and so on) and a minority showing the opposite bias [17]. The very existence of such a minority, such as left-handers in humans, is an issue in evolutionary biology. In fact, for humans, it has been claimed that it could be associated with frequency-dependent advantages in fighting [22], though the evidence is controversial [23].

A general framework for explaining the evolution of brain asymmetry at the population level has been proposed by Ghirlanda & Vallortigara [24]. They made use of games theory to show that alignment of the direction of behavioural asymmetries in a population can arise as an evolutionarily stable strategy (ESS), when individually asymmetrical organisms must coordinate their behaviour with that of other asymmetrical organisms. In this model, frequency-dependent selection, e.g. prey–predator interaction, would emerge spontaneously as an ESS without any specific association to a fighting hypothesis.

The model has been extended by considering intraspecific interactions [25], i.e. selective pressures of synergistic (cooperative) and antagonistic (competitive) interactions on individuals being lateralized in the same or opposite direction within the same species. Ghirlanda *et al*. ] [25] introduced a fitness function, f(X)=a(X)+cs(X), where a(X) accounts for antagonistic interaction (say a competition function) and *s*(*X*) for synergistic interaction (say a cooperation function); *c* is the control parameter tuning the relative strength between a(X) and s(X). The variable X is given by the ratio between L and R. The model shows, in an almost fully analytical way, that under proper conditions, there exists an unequal number of equilibrium of left- and right-lateralized individuals.

Although the theory has sometimes been interpreted in terms of a ‘social hypothesis’, i.e. that population-level lateralization would emerge in social species (see [17] and comments therein), it is much more general than that [26]. The ESS hypothesis can predict either population- or individual-level lateralization, depending on the type of interactive behaviour considered [27] (e.g. cooperative or competitive) and its ecological context. Although the advantage of being aligned in the same direction is clear in cooperative behaviour, it is not so for predatory interactions. It may be more advantageous for a predatory act to not be directional since population-level bias would be accompanied by predictability, and if an individual attacks a prey, it would be more convenient for it to be unpredictable. As a consequence, although each individual would have an (individual-level) bias because of the advantages (mentioned above) for the machinery of the brain of being lateralized, there will be 50 : 50 right- and left-biased individuals in the population. This has been proved to be the case in some predator species, such as sailfish, which are lateralized at the individual level in attacking schooling sardines on one side (and the stronger they are lateralized, the more successful they are at capturing their prey), but that overall does not show a population-level bias [28].

Developments of the theory have been proposed, for example, Tonello & Vallortigara [29] used a more numerical approach to show that simulation confirms the behaviour of the previous model in a detailed way and, in particular, they showed that whatever the initial proportion between L and R, it will converge to the predefined equilibrium imposed by the c parameter: considering a group of individuals (large enough), whatever the L/R initial ratio, it will always evolve to the ratio defined by c, the relative strength between competition and cooperation. Abrams & Panaggio [30] also developed a mathematical model to test the idea that population-level hand preference represents a balance between selective costs and benefits arising from cooperation and competition.

Other theories have been proposed as well to explain population-level asymmetry, though they seem to have been less successful in accounting for the generality of the available evidence (e.g. theories based on features of inter-hemispheric transfer such as timing [31]).

What is lacking, however, in theorization about the evolution of brain asymmetry is an overarching general hypothesis that may subsume all different aspects of current models. Here, we tried to provide an overarching general framework based on the minimization principle of the energy and of the free energy, which proved so valuable in other areas of neurosciences [32–36].

Brain studies are indeed intimately related to free-energy considerations. Brains are ‘open’ systems, continuously exchanging energy with their environment. Symmetry under time translations is broken for the brain and biological systems, which means that the origin of the time axis (the time of their birth) cannot be translated; they are *ageing* systems. The entropy controls their time evolution, which is therefore irreversible, i.e. time-reversal symmetry is also broken; *the arrow of time* pointing in the increasing entropy direction thus arises. These features are naturally accounted for by considering the free energy [32–34], whose behaviour also provides a dynamic framework to the discussion on symmetries and asymmetries (see [37]), and their interplay with evolutionary pressure [38]. Moreover, the free-energy concept and formalism are useful in neuroscience to study the structures of sensory inputs in relation to Bayesian action planning and learning in the action-perception cycle [34–36,39,40].

## 2. Individual-level asymmetry

Consider a system ψ that can be in two states, $\psi_{R}$ or $\psi_{L}$, corresponding to two different functional behaviours, e.g. let ψ denote one of the brain hemispheres, the left or the right one. In full generality, in standard matrix notations and calculations, in the space of two-component vectors, we may describe it by the doublet:


(2.1) $$\psi =\left(\begin{array}{c} \psi_{R}\\ \psi_{L} \end{array}\right)\;,$$

and consider the basis of the 2×2 matrices $\tau_{i}\equiv (1/2)\sigma_{i}$, i=1,2,3, with $\sigma_{i}$ the Pauli matrices given in appendix A. There the anticommutation and commutation relations for the $\tau_{i}$, i=1,2,3, matrices are reported, and the matrices $\tau_{\pm }=\tau_{1}\pm i\tau_{2}$ and their commutation relations are also introduced.

We consider now two elements $\psi^{1}$ and $\psi^{2}$ and the corresponding matrices ${\bar{\tau }}^{1}$ e ${\bar{\tau }}^{2}$. For the two-component total system, we have to consider the tensor product $\psi^{1}\times \psi^{2},\;$ with ${\bar{\tau }}_{tot}={\bar{\tau }}^{1}+{\bar{\tau }}^{2}$. One has, up to normalization constants, the triplet of states:


(2.2) $$\psi_{R}^{1}\psi_{R}^{2}\;,$$

(2.3) $$\left(\psi_{R}^{1}\psi_{L}^{2}+\psi_{L}^{1}\psi_{R}^{2}\right)\;,$$

(2.4) $$\psi_{L}^{1}\psi_{L}^{2}\;,$$

corresponding to $s_{3,tot}=+1,0,-1$, respectively, and $s_{tot}=1$, and the singlet state with $s_{3,tot}=0$ and $s_{tot}=0$


(2.5) $$\begin{array}{ccc} & (\psi_{R}^{1}\psi_{L}^{2}-\psi_{L}^{1}\psi_{R}^{2}). & \end{array}$$

Note that the triplet states ($s_{tot}=1$) are symmetric under R↔L exchanges; on the contrary, the singlet state ($s_{tot}=0$) is anti-symmetric (the quantity *S_tot_* is introduced in appendix A).

The energy is a scalar quantity, i.e. it is invariant under rotation transformations induced by $\tau_{i}$. It is, therefore, dependent on such matrices only through scalars obtained by them, i.e. of type $g\;{\bar{\tau }}^{1}\cdot {\bar{\tau }}^{2}$, with constant g.

In appendix A, we show that ${\bar{\tau }}^{1}\cdot {\bar{\tau }}^{2}=-3/4$ for the singlet, while ${\bar{\tau }}^{1}\cdot {\bar{\tau }}^{2}=+1/4$ for the triplet case, and therefore, the antisymmetric singlet configuration realizes the lowest energy state of the system. The energy gap between the two configurations is 1 (in our dimensionless units).

In conclusion, the result is that the lateralization (asymmetry) of the total system finds its origin in the dynamic principle of minimization of the energy.

Any realization of lateralization at the biomolecular and cellular level in specific organs, subjects and species is allowed, provided that such a minimum energy requirement is satisfied. The biochemical and cellular realizations are the manifestations, not the cause of the lateralization. The result provides an example of how the underlying physical dynamics may act as a ‘waveguide’ for the biochemical and cellular activity in a biological system.

We also notice that the obtained result is not ‘in the average’, it is not of statistical origin. The system must be asymmetric to be in its minimum energy state, which guarantees its stability. This reminds us of the Schrödinger observations in *What is life?* [41] where he remarks that *regularities only in the average* (p. 78) emerging from statistical mechanisms are not enough to explain the *enigmatic biological stability* (p. 47).

## 3. Population-level asymmetry

In our analysis at the group level, we show that the spontaneous emergence of directional asymmetry in a population arises as a consequence of the minimization of the free energy of the system which guarantees its stability equilibrium.

We consider the ‘global’ state of a population of N components, each one of them with a definite lateralization R or L. It is useful to use the vector notation |R⟩, |L⟩, with orthonormalization conditions ⟨R|R⟩=1=⟨L|L⟩ and ⟨L|R⟩=0=⟨R|L⟩.

Let b and $b^{†}$ be matrices characteristic of some environment agent coupled with the set of elements of our system. The interaction might be representable as a contribution to the system energy of the type $H_{I}\propto \lambda (b^{†}\tau_{-}\;+\;b\tau_{+})$, where λ is a convenient coupling constant and the τ matrices are given in equations (A 9) and (A 10).

For the population of $N=N_{R}+N_{L}$ elements, we denote by $|\ell \rangle_{X}$ the normalized superposition of a number ℓ of |R⟩-states and a number (N−ℓ) of |L⟩ states, with the ratio $X=N_{L}/N_{R}$. The expression for the state $|\ell \rangle_{X}$ is given by equation (A 12) in appendix A.

A measure of the asymmetry $N_{L}\neq N_{R}$, i.e. X≠1 is given by


(3.1) $$\begin{array}{ccc} & M\equiv {,}_{X}\langle \ell |\tau_{3}|\ell \rangle_{X}=\ell -\frac{1}{2}N=\frac{1}{2}(N_{R}-N_{L}), & \end{array}$$

which can be also written as $M=M_{X}=(1/2)N_{R}(1-X)$ and M≠0 indeed signals asymmetry under exchanges R↔L. The value of X≷1 determines the sign of M.

Note that the quantity M describes a global (*collective*) property of the system, not of its individual components.

The analysis reported in appendix A shows that the asymmetry described by the non-zero value of M produces the change SU(2)→E(2) of the transformation properties of the population system. The physical meaning of these mathematical features is that, for large N, the population of N individual components is ‘locally felt’ by the single component as a whole *collective* agent.

We now show that the domain of values acquired by the ratio X is determined by the condition of the minimization of the free energy, which formally expresses the condition for the stability of the system.

It is convenient to put


(3.2) $$\begin{array}{ccc} & \frac{N_{L}}{N}=a,\;\frac{N_{R}}{N}=1-a, & \end{array}$$

and since a+(1−a)=1, in full generality we may set $a=\tanh^{2}\;\theta$ and $1-a=1/\;\cosh^{2}\;\theta$, so that we obtain $N_{L}/N_{R}=a/(1-a)=\sinh^{2}\;\theta$, i.e. $X=X(\theta )=\sinh^{2}\;\theta$ (and M=M(θ)).

The ratios $N_{R}/N$ and $N_{L}/N$ give of course the probability of getting one R-state and one L-state, respectively, out of N independent possibilities.

The probability to get one R-state and a number of Xs of L-states is


(3.3) $$W_{X}\equiv \frac{N_{R}}{N}{\left(\frac{N_{L}}{N}\right)}^{X}=\frac{1}{\cosh^{2}\theta }\tanh^{2X}\theta ,$$

with $\sum_{x}W_{X}=1$ and $0<W_{X}<1$ for non-zero θ. For notational simplicity X denotes X(θ). Putting $S_{X}=-\ln W_{X}\;$, we have


(3.4) $$S_{X}=-(\sinh^{2}\theta \ln\sinh^{2}\theta -\cosh^{2}\theta \ln\cosh^{2}\theta ).$$

We recognize that $S_{X}$ is the entropy of the population [42] (a population of N individuals may access many configurations, e.g. labelled by different values of the X ratio; the higher the number of accessible configurations, the higher the entropy). The free energy is given by:


(3.5) $$F=H_{X}-\frac{1}{\beta }S_{X},$$

where $H_{X}=XE_{X}$ is the energy associated with the configuration with the X ratio, and $\beta =1/k_{B}T$. According to thermodynamic principles [43,44], the system stability requires the minimization of F: dF=0. This gives


(3.6) $$-\beta E_{X}=\ln\tanh^{2}\theta =\ln\frac{N_{L}}{N},$$

where equation (3.2) has been used in the second equality. We see theractions on individuals being lateralizat, as expected on general thermodynamic grounds, the minimization of free energy leads to values of $N_{L}$ proportional to the Boltzmann distribution factor, $N_{L}=Ne^{-\beta E_{X}}$. More specifically, equation (3.6) shows that $N_{L}\neq N$, i.e. $N_{R}\neq 0$, since $N_{L}/N=1$ would imply $\beta E_{X}\approx 0$, i.e. the physically unrealistic value of T≈∞ and/or $E_{X}\approx 0$ should be reached. The result (equation (3.6)) also implies that $N_{L}\neq 0$, i.e. $N_{R}\neq N$ since for $N_{L}/N=0$ it should be $\beta E_{X}\approx \infty$, implying the unphysical value T≈0 and/or $E_{X}\approx \infty$.

Summing up, the derived result is that $N_{L}\neq 0$, $N_{R}\neq 0$, and thus $X=N_{L}/N_{R}$ is a non-zero finite quantity. The specific value of X depends on the value that $\beta E_{X}$ may acquire in specific boundary conditions.

We remark that the symmetric distribution $N_{L}=N_{R}$, i.e. X=1 (M=0), or, equivalently, $N_{L}/N=1/2$, i.e. $\beta {E_{X}}_{{|}_{X=1}}=\ln\;2$ (from equation (3.6)), is excluded by applying also in this case, the derivation used in §2. In the present case, we denote by $\Psi^{1}$ and $\Psi^{2}$ the two populations R and L. Again, the fact that the energy $E_{X}$ is a scalar quantity and the computation similar to the one in §2 show that the equilibrium stability of the global population system (granted by its lowest energy) is realized by the antisymmetric singlet configuration X≠1. The occurrence of a symmetric L/R distribution (e.g. the existence of functional Bilateria in the pre-Cambrian era) would denote the ‘dynamical necessity’ of the evolution towards the stability of the antisymmetric configuration.

Note that, reciprocally, given the ratio X(θ), equation(3.6) may be taken to ‘define’ the system temperature T.

## 4. Discussion

We have shown in §2 that at the level of the individual component, e.g. the two sides of the brain, the configuration that is asymmetric under R↔L exchanges is the one minimizing the energy, and it is, therefore, the one realized as the stability configuration. In its derivation, we have used solely matrix calculations and the scalar property of the energy, namely its invariance under rotation transformations. Of course, provided that the physical requirement of minimum energy is satisfied, one may have a rich diversity of molecular realizations of the R−L asymmetry, depending on the considered specific biological system. The various associated phenomenological processes (e.g. game theory processes [24,25], ESS, issues in evolutionary biology, social hypothesis, cooperation/competition strategies, etc.) may be thought of as the possible ‘manifestations’ of the general physical principle of energy minimization generating the R−L asymmetry. These processes have their origin in R−L asymmetry, are not its cause. The individual R−L asymmetry appears thus grounded on a physical dynamic basis, not on empirical occasional occurrences. This point may be the object of further physiological and functional analysis related to evolutionary issues [20] that we leave to future work.

We have then considered in §3 a population of N individual components. In our algebraic analysis, we have shown that energy and free-energy minimization, which guarantee the stability and equilibrium of the population, is realized by the configuration antisymmetric under R↔L exchanges. Moreover, we have shown that the population system at large N behaves as a ‘collective’ system.

In particular, we have that in the limit of large N the terms $S_{+}S_{-}/N$ under the square roots in equation (A 16) become negligible and the change of the algebra su(2)→e(2) occurs. The physical meaning of these mathematical aspects is that the population of large N components is observed ‘locally’ by the single individual component as the environment acting as a whole *collective* agent. This amounts to saying that correlations among the individual components dynamically arise as a consequence of the asymmetry M≠0 (the breakdown of the rotational SU(2) symmetry). M indeed describes a collective property structuring the population *as a whole*.

We observe that the change SU(2)→E(2) signals the *dynamical rearrangement* of the SU(2) symmetry structures to the E(2) ones. A process leading from *form-to-form* (a morphogenetic process) is at work. From a phenomenological standpoint, ‘new’ symmetry structures (the ones of E(2)) appear to emerge with a higher degree of ‘simplicity’ (a lower degree of complexity) [37]: the axial (one-parameter cylindrical) rotations of E(2) *vs* the SU(2) spherical (three-parameter) rotations. However, such a spontaneous emergence of symmetry and simplicity from the evolution processes does not describe the whole story. The analysis of the mathematical structure of the process shows indeed that the dynamical rearrangement of symmetry does preserve the number of the transformation parameters: E(2) is a three-parameter group, just as SU(2). As shown in appendix A, besides the one-parameter cylindrical rotations, the two ‘translations’ $N_{R}\to N_{R}\pm 1$ and $N_{L}\to N_{L}\mp 1$ also belong to E(2) (cf. equation (A 14)). They produce changes in the *collective* properties of the population system (see equation (3.1)), appearing at the phenomenological level triggered by some ‘evolutionary pressure’ [38] or environmental perturbations inducing the system to self-tune again to free-energy minimization (see below and appendix A). This will also offer the possibility to test in studies on large populations of individuals the interplay between the appearance of symmetry and simplicity features and changes in the collective population behaviours.

We note that in the large population limit N→∞, the θ parameter introduced in the previous section may be well considered ranging in a continuous interval of values, thus allowing its *fine-tuning* and consequently the *fine-tuning* of the $\beta E_{X}$ values (cf. equation (3.6)), for the stability system state.

In the large N limit, the contribution $H_{I}$ to the system energy introduced in §3 becomes $H_{I}\propto \lambda \sqrt{N}(b^{†}S_{-}\;+\;bS_{+})$ (see equation (A 17)), where the original coupling constant λ gets multiplied by $\sqrt{N}$. We have thus the transition to the strong coupling regime, $\lambda \sqrt{N}\gg \lambda$. Remarkably, the amount of energy (corresponding to $H_{I}$) is stored in the ‘correlations’ thus established among individuals.

Our analysis provides thus the mathematical description of the fact that ‘...Of course, the individual does not have a direct measure of how many left- or right-lateraled individuals are there in the population...‘ [29] and our answer to the question: ‘...will an individual meet a large enough number of others so that the L/R proportion will be in line with the whole population?‘ [29] is negative. The individual, as already mentioned, sees ‘locally’ the population of (a large number) N components, as a ‘whole’ collective system, not as a ‘collection’ of individuals, but rather as a form of ‘social interaction’ [45], as a sort of ‘... environmental effects...‘ [29]. We have provided the formal proof that through collective modes $S_{\pm }$ in the system state $|\ell \rangle_{X}$, ‘...[communication of each individual is] in an indirect way through environmental change in a social way, as a form of stigmergy...‘ [29,45–47].

The individual ‘expectation’ is that ‘...under proper conditions, there exists an unequal number of L and R lateralized individuals’ [29], which, as observed in §3, is proportional to the Boltzmann factor (cf. equation (3.6)), consistently with general physical principles.

We have also shown (in appendix A) the stability under perturbing actions on the ratio $X=\sinh^{2}\;\theta$ collectively characterizing the population. By minimizing the free energy, the population system may ‘tune’ itself to face environmental perturbations affecting the ratio $X\to X^{\prime }$, with the result of re-adjustment in the θ-parameter space to the equilibrium $\theta^{\prime }=\theta$ ($X^{\prime }\to X$). This provides, on a theoretical basis, the justification of the numerical result that the ratio $X^{\prime }$ converges to the predefined equilibrium value X [29].

Our result thus supports the observation that [29] ‘...the system self-organizes in an intelligent way (i.e. *dynamical* way, in our present description) because it moves autonomously towards an optimal equilibrium—so it is intelligent only when operating as a group’, namely, as we have found, it behaves as a collective system.

## Data Availability

This article has no additional data.

## Appendix A

We define

(A 1)
$$\psi (R)=\left(\begin{array}{c} \psi_{R}\\ 0 \end{array}\right),\;\psi (L)=\left(\begin{array}{c} 0\\ \psi_{L} \end{array}\right)$$

The Pauli matrices and the 2×2 identity matrix 1I are:

(A 2)
$$\sigma_{1}=\left(\begin{array}{cc} 0 & 1\\ 1 & 0 \end{array}\right),\;\sigma_{2}=\left(\begin{array}{cc} 0 & -i\\ i & 0 \end{array}\right),\;\sigma_{3}=\left(\begin{array}{cc} 1 & 0\\ 0 & -1 \end{array}\right),\;1\;I=\left(\begin{array}{cc} 1 & 0\\ 0 & 1 \end{array}\right).$$

The matrices $\tau_{i}\equiv (1/2)\sigma_{i}$, i=1,2,3, anticommute

(A 3)
$$\{\tau_{i},\tau_{j}\}=\tau_{i}\tau_{j}+\tau_{j}\tau_{i}=\frac{1}{2}1\;I\delta_{ij},\;i,j=1,2,3,$$

with $\delta_{ij}=0$ for i≠j, and $\delta_{ij}=1$ for i=j, and $\tau_{i}^{2}=1/4$ for any i since $\sigma_{i}^{2}=1$. The commutators are:

(A 4)
$$\begin{array}{ccc} & [\tau_{i},\tau_{j}]=\tau_{i}\tau_{j}-\tau_{j}\tau_{i}=i\epsilon_{ijk}\tau_{k},\;i,j,k=1,2,3. & \end{array}$$

The matrices $\tau_{\pm }=\tau_{1}\pm i\tau_{2}$ are also defined:

(A 5)
$$\begin{array}{ccc} & \begin{array}{c} \tau_{+}=\left(\begin{array}{cc} 0 & 1\\ 0 & 0 \end{array}\right),\;\tau_{-}=\left(\begin{array}{cc} 0 & 0\\ 1 & 0 \end{array}\right), \end{array} & \end{array}$$

with

(A 6)
$$[\tau_{-},\tau_{+}]=-2\tau_{3},\;[\tau_{3},\tau_{\pm }]=\pm \tau_{\pm }.$$

It is $\tau^{2}=\tau_{1}^{2}+\tau_{2}^{2}+\tau_{3}^{2}$. It commutes with all the $\tau_{i}$, i=1,2,3, $[\tau^{2},\tau_{i}]=0$. The eigenvalue equations are:

(A 7)
$$\begin{array}{lll} {\bar{\tau }}^{2}\psi (R) & = & s(s+1)\psi (R)=\frac{3}{4}\psi (R) \end{array},$$

(A 8)
$$\begin{array}{ccc} & \begin{array}{ccc} \tau_{3}\psi (R) & = & s_{3}\psi (R)=\frac{1}{2}\psi (R) \end{array} & \end{array}$$

with s=1/2. Similar relations hold for ψ(L), with $s_{3}=-(1/2)$.

The composition of two $\bar{\tau }$s gives ${\bar{\tau }}_{tot}^{2}=({\bar{\tau }}^{1}{)}^{2}+({\bar{\tau }}^{2}{)}^{2}+2{\bar{\tau }}^{1}\cdot {\bar{\tau }}^{2}$, with eigenvalue $s_{tot}(s_{tot}+1)$. We have then for the scalar ${\bar{\tau }}^{1}\cdot {\bar{\tau }}^{2}=(1/2)[s_{tot}(s_{tot}+1)-s_{1}(s_{1}+1)-s_{2}(s_{2}+1)]$, where $s_{1}=s_{2}=1/2$.

In equations (2.2) - (2.4) and (2.5) the triplet and the singlet states have been introduced. For the singlet $s_{tot}=0$ and thus ${\bar{\tau }}^{1}\cdot {\bar{\tau }}^{2}=-3/4$. For the triplet $s_{tot}=1$ and then ${\bar{\tau }}^{1}\cdot {\bar{\tau }}^{2}=1/4$.

The algebra (A 4) and (A 6) is the one of the SU(2) rotation group for the doublet ψ.

When we consider a population of N individuals, as introduced in §3, we may write the τ matrices as

(A 9)
$$\begin{array}{lll} \tau_{3,i} & = & \frac{1}{2}(|R\rangle_{ii}\langle R|-|L\rangle_{ii}\langle L|) \end{array},$$

(A 10)
$$\begin{array}{ccc} & \begin{array}{ccc} \tau_{+,i} & = & |R\rangle_{ii}\langle L|,\;\tau_{-,i}=|L\rangle_{ii}\langle R|, \end{array} & \end{array}$$

with i=1,2,...N, and

(A 11)
$$\begin{array}{ccc} & \tau_{3}=\sum_{i=1}^{N}\tau_{3,i},\;\tau_{\pm }=\sum_{i=1}^{N}\tau_{\pm ,i}, & \end{array}$$

satisfying for each i=1,2,...N the su(2) rotational algebra (equation (A 6)).

The normalized superposition $|\ell \rangle_{X}$ of ℓ|R⟩-states and (N- ℓ) |L⟩ -states, for a given ratio $X=N_{L}/N_{R}$ is given by [48,49]:

(A 12)
$$|\ell \rangle_{X}={\left(\begin{array}{l} N\\ \ell \end{array}\right)}^{-\frac{1}{2}}(|L\rangle_{1}...|L\rangle_{N-\ell }|R\rangle_{N-\ell +1}...|R\rangle_{N}+...+|R\rangle_{1}....|R\rangle_{\ell }|L\rangle_{\ell +1}...|L\rangle_{N}).$$

Note that $|\ell \rangle_{X}$ is not eigenstate of $\tau_{\pm }$:

(A 13)
$$\tau_{+}|\ell \rangle_{X}=\sqrt{\ell +1}\sqrt{N-\ell }|\ell +1\rangle_{X^{\prime }},\;\tau_{-}|\ell \rangle_{X}=\sqrt{N-(\ell -1)}\sqrt{\ell }|\ell -1\rangle_{X^{\prime \prime }},$$

with $X^{\prime }$ and $X^{\prime \prime }$ denoting the values of Xs from the changes ℓ→ℓ±1, respectively.

By introducing $(S_{-}{)}^{†}=S_{+}$, with $[S_{-},S_{+}]=1$, and

(A 14)
$$S_{+}|\ell \rangle_{X}=\sqrt{\ell +1}|\ell +1\rangle_{X^{\prime }},\;S_{-}|\ell \rangle_{X}=\sqrt{\ell }|\ell -1\rangle_{X^{\prime \prime }},\;S_{+}S_{-}|\ell \rangle_{X}=\ell \;|\ell \rangle_{X},$$

according to equations (3.1) and (A 13) $\tau_{3}$ and $\tau_{\pm }$ are represented on $|\ell \rangle_{X}$ as

(A 15)
$$\begin{array}{ccc} & \begin{array}{ccc} \tau_{3} & = & S_{+}S_{-}-\frac{1}{2}N, \end{array} & \end{array}$$

(A 16)
$$\frac{\tau_{+}}{\sqrt{N}}=S_{+}\sqrt{1-\frac{S_{+}S_{-}}{N}},\;\frac{\tau_{-}}{\sqrt{N}}=\sqrt{1-\frac{S_{+}S_{-}}{N}}S_{-}.$$

We remark that $\tau_{3,\pm }$ in equations (A 15) and (A 16) still obey the su(2) algebra. From (A 16) we have

(A 17)
$$\begin{array}{ccc} & \frac{\tau_{\pm }}{\sqrt{N}}\to S_{\pm },\;\mathrm{for}N\gg \ell , & \end{array}$$

and, putting $S_{3}\equiv \tau_{3}$, we have for N≫ℓ

(A 18)
$$\begin{array}{ccc} & [S_{-},S_{+}]=1,\;[S_{3},S_{\pm }]=\pm S_{\pm }, & \end{array}$$

which shows that in the limit of large N, only the commutators with $S_{3}$ in the original su(2) algebra (A 6) are preserved, namely rotations around the three axes. Rotations around the ‘± axes’ (i.e. one and two axes) are substituted by ‘translations’ of ℓ→ℓ±1, changing the number $N_{R}\to N_{R}\pm 1$ and $N_{L}\to N_{L}\mp 1$, respectively (see the first two equations in (A 14)), i.e. M→M∓1. This means that in the large N limit it is possible to operate changes on the *collective* properties (e.g. on the M values) of the population system *as a whole* (see equation (3.1)). The population state $|\ell \rangle_{X}$ is represented as being made of (‘containing’) a number ℓ of $S_{+}S_{-}$ modes, ${,}_{X}\langle \ell |S_{+}S_{-}|\ell \rangle_{X}=\ell$ (see (A 14)) describing its collective structure.

Note that we assume the thermodynamic limit by which large N implies large volume V, so to have finite density. In such a sense, the large N limit is equivalent to the large volume limit.

We just mention that in the language of group theory the algebra equation (A 18) is the one of the E(2) group and the process SU(2)→E(2) for N→∞ is called group contraction [50,51].

Consider now a perturbation affecting the ratio value $X\to X^{\prime }$, so that $W_{X}\to W_{X^{\prime }}$ with

(A 19)
$$S_{X^{\prime }}=-\ln W_{X^{\prime }}=-(X^{\prime }\ln\sinh^{2}\theta -(X^{\prime }+1)\ln\cosh^{2}\theta ).$$

By minimizing the free energy with respect to θ, the population system ‘tunes’ itself to face the perturbation. This amounts to searching for an equilibrium state in the space of the θ-parameter. Taking the derivative with respect to θ and putting it equal to zero shows that the equilibrium is obtained at $\sinh^{2}\;\theta^{\prime }=\sinh^{2}\;\theta$ and $W_{X^{\prime }}=W_{X}$, i.e. the equilibrium is re-established for $\theta^{\prime }=\theta$.
